# Effect of exogenous surfactant on Paediatric Bronchoalveolar lavage derived macrophages’ cytokine secretion

**DOI:** 10.1186/s12890-019-1006-4

**Published:** 2019-12-05

**Authors:** Lyné van Rensburg, Johann M. van Zyl, Johan Smith, Pierre Goussard

**Affiliations:** 1 Division of Clinical Pharmacology, Department of Medicine, Faculty of Medicine and Health Sciences Stellenbosch University, PO Box 241, 8000; Francie van Zijl Drive, Cape Town, Tygerberg 7505 South Africa; 2 Department of Pediatrics, Tygerberg Children’s Hospital, Faculty of Medicine and Health Sciences, Stellenbosch University, Tygerberg, 7505 South Africa

**Keywords:** Bronchoscopy, Bronchoalveolar lavage, Alveolar macrophages, Cytokines

## Abstract

**Background:**

Bronchoalveolar lavage is a useful bronchoscopy technique. However, studies in “normal” children populations are few. Furthermore, the anti-inflammatory effects of exogenous pulmonary surfactants on the bronchoalveolar cellular components are limited.

**Methods:**

Thirty children, aged 3 to 14 years, underwent diagnostic bronchoscopy and bronchoalveolar lavage. Differential cytology, cytokine and chemokine measurements were performed on the fluid after exogenous surfactant exposure. The aim of the study was to investigate the potential anti-inflammatory effects of exogenous surfactants on the bronchoalveolar lavage fluid, specifically alveolar macrophages of healthy South African children.

**Results:**

Alveolar macrophages were the predominant cellular population in normal children. Patients with inflammatory pneumonopathies had significantly more neutrophils. Levels of inflammatory cytokines were significantly lower after exogenous surfactant exposure. Moreover, IL-10 and IL-12 cytokine secretion increased after exogenous surfactant exposure.

**Conclusion:**

This study provides the first data on bronchoalveolar lavage of healthy South African children. Bronchoalveolar lavage fluid from patients with pulmonary inflammation was characterised by neutrophilia. Finally, we propose that exogenous surfactant treatment could help alleviate inflammation in diseased states where it occurs in the tracheobronchial tree.

## Background

Bronchoalveolar lavage (BAL) is a procedure indicated for children presenting with several inflammatory and non-inflammatory lung conditions where a sample of the endobronchial environment is taken. This allows for the furthering knowledge regarding paediatric pneumonopathies [1]. There are many restrictions regrading BAL for research purposes, particularly for healthy to normal BAL cellular and non-cellular constituents.

The aim of the present study was to 1) to assess the cellular components in BALs of children with inflammatory and non-respiratory problems, and 2) some of the common inflammatory mediators and to establish whether the synthetic pulmonary surfactant Synsurf® elicits immunomodulatory and immunogenicity characteristics in comparison to the natural derived surfactants, Curosurf® and Liposurf®, currently used today. For this purpose, we investigated cytokine production, of BAL derived alveolar macrophages (AMs) treated with either Synsurf®, Curosurf® or Liposurf®.

## Methods

The study was performed under approval from the Ethical Review Committee of the Faculty of Health Sciences of Stellenbosch University (Reference N13/07/099). After written informed consent, bronchoalveolar sampling for this study were collected from children deemed not to have infectious conditions (i.e. Tuberculosis or HIV). Samples where from children with structural problems that underwent bronchoscopy for diagnostic reasons. These were healthy children with upper airway obstruction or chronic stridor without evidence of parenchymal disease on their X-rays or acute infection. Bronchoscopy was only done in these children when they were healthy and had no respiratory symptoms during the two weeks before the bronchoscope procedure. BAL sample collection was performed after topical anaesthesia with 4010 lidocaine spray. A flexible fibre optic bronchoscope was introduced through the upper airway and wedged in a segment of the right middle lobe. One 4–8 ml of BAL aliquot per patient was aspirated with gentle hand suction and collected for this study. A total of 30 paediatric patients’ BAL samples were collected.

### Surfactant preparations

Synsurf® was prepared as described previously by van Zyl et al. [2]. The natural surfactants used were Curosurf®, a porcine lipid extract surfactant suspension (Chiesi Farmaceutici SpA) and Liposurf®, a bovine lipid extract surfactant suspension (Cipla).

### Isolation of alveolar macrophages

AMs from bronchoalveolar lavage samples were isolated from other contaminant cell types by Histopaque-1077 (Sigma, St. Louis, MO) density gradient centrifugation of whole BAL aspirates. After isolation the cells were resuspended in RPMI 1640 (Roswell Park Memorial Institute media) culture medium supplemented with 10% fetal calf serum, 1% l-glutamine solution (200 mM), and 1% Penicillin-Streptomycin. After cell counting, AMs samples were allowed to adhere in 6 well flat bottom cell culture plates maintained in a humidified 5% CO_2_–95% atmospheric air incubator at 37 °C. To ensure macrophage survival and adherence, the cells were supplemented with up to 25% fetal calf serum in the beginning. This was usually refreshed only once thereafter at the same concentration followed by 10% supplementation as indicated above. Cells were left overnight to attach to the culture plates. All nonadherent cells that floated were regarded as “non-viable” and were “washed” off. Only adhered macrophages were considered as alive (used in the experiments after ~ 12 h). This procedure possibly also helped to separate lymphocytes from the population. Cell viability was assessed by trypan blue exclusion before conducting each experiment. Equal parts of 0.4% trypan blue dye was added to the cell suspension to obtain a 1 to 2 dilution before cell count. Live cells with intact cell membranes are not coloured however, if cells take up trypan blue, they are considered non-viable. Viability was established at ~ 85–90%. Specific care was taken upon harvesting of adherent cells to minimise inadvertent activation.

### Inflammatory cytokines

To evaluate the anti-inflammatory effects of exogenous surfactants, Curosurf®, Liposurf® and Synsurf® were standardised to equivalent phospholipid concentrations, 500–1500 μg/ml and incubated with LPS- (1 μg/ml) stimulated BAL-derived AMs over 24 h. Control samples consisted of LPS-stimulated AMs samples devoid of any surfactant. Cytokines production in the alveolar macrophage supernatants were analysed by using a multiplex (V-PLEX) human cytokines’ electrochemiluminescence-based ELISA kit (Meso Scale Discovery®) as per manufacturer’s instructions. All samples were run in duplicate, and the mean values were used for statistical analysis. Values were calculated in picogram cytokine per milliliter (pg/ml) of sample. Cytokines screened for were: IL-1β, IL-2, TNF-α, IL-6, IL-8, INF-γ, GM-CSF, IL-10, IL-12.

## Results

**Fig. 1:**
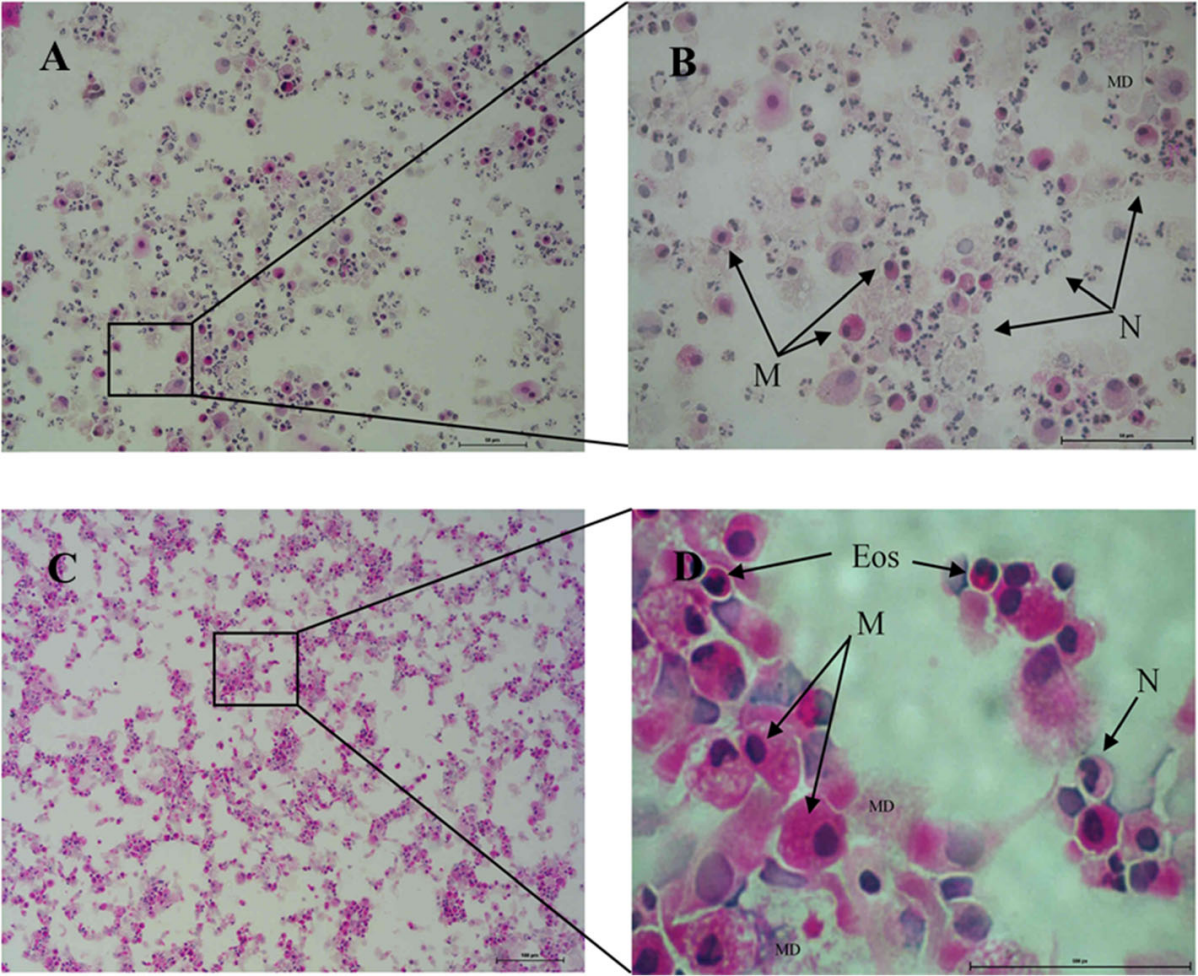
H&E staining of human BAL sample after mononuclear cell isolation from patient diagnosed with asthma (**a** & **b**) and a healthy patient with an airway obstruction (**c** & **d**); Abbreviations: M: Macrophage, N: Neutrophil, Eos: Eosinophil, MD: Mucus debris. Scale bar represents: (**a** & **b**) 50 μm, (**c**) 100 μm, (**d**) 500 px

As shown in Fig. 1, the representative haematoxylin and eosin (H&E) staining indicate different cell populations found in BAL samples. Figure 1a and b demonstrated a larger population of neutrophils indicating neutrophilia. These populations were excluded from the current study as our focus was on macrophage initiated cytokine release. Figure 1c and d indicates the optimal cell population that was used as a standard for the appropriation of the study.

During our experiments to assess the release of cytokines by LPS-stimulated AMs as well as in the presence of surfactants, we were mindful that during the enrichment and handling processes of these cells, disturbance of the original milieu as well as even the transfer process to cell culture plates could be modulatory on cytokine production. Table 1 represent the results of stimulated macrophage secretion of the nine inflammatory cytokines we studied. Comparison of the different surfactants with regards to inflammatory and anti-inflammatory cytokine secretion did not reveal significant differences; therefore, data representation is more effective within descriptive statistical analysis.

**Table 1:** The mean, SD (standard deviation), P25 (25th percentile), P50 (median), & P75 (75th percentile) of LPS (1 μg/ml)-stimulated cytokine production in BAL-derived human alveolar macrophage supernatant concentrations measured at 24 h in the presence of surfactants. Control samples - LPS –stimulated AMs

	Control	Curosurf®	Liposurf®	Synsurf®
IL-1β (pg/ml)				
Mean	176.45	168.87	118.01	97.6
SD	305.32	292.62	210.07	166.01
P25	0.09	0.3	0.34	0.27
P50	0.25	0.39	1.29	0.6
P75	529	495.5	300.5	332
IL2 (pg/ml)				
Mean	7.61	5.16	4.38	10.91
SD	1126	7.77	5.84	19.03
P25	0.017	0.14	0.22	0.14
P50	0.0722	0.38	0.022	0.31
P75	1951	7.25	8.86	27.45
IL6 (pg/ml)				
Mean	650.4	452.78	321.08	265.7
SD	1126	815.29	634.03	412.02
P25	0.017	0.17	0.07	0.14
P50	0.0722	0.29	0.18	0.24
P75	1951	1134	341	759.5
IL8 (pg/ml)				
Mean	3588	3193.71	3139.73	3101.79
SD	6020	5153.64	5081.01	4997.25
P25	55.35	89	84.5	95.75
P50	169	249.5	251	198.5
P75	10,540	10,532.5	10,316.5	10,273
TNF-α (pg/ml)				
Mean	462	120.73	110.01	56.87
SD	–	195.09	181.01	99.96
P25	–	0.08	0.1	0.08
P50	–	0.13	30.98	0.13
P75	–	273.5	219.93	95.35
INF-γ (pg/ml)				
Mean	5.771	1.93	3.06	1.12
SD	8.039	2.5	4.8	0.85
P25	0.0864	0.28	0.45	0.55
P50	5.771	0.49	0.82	0.6
P75	11.46	2.57	5.68	1.75
GM-CSF (pg/ml)				
Mean	114.1	84.5	61.89	62.33
SD	197.3	157.67	129.1	107.94
P25	0.1176	0.28	0.21	0.06
P50	0.2565	0.35	0.42	0.22
P75	342	183	86.3	184
IL10 (pg/ml)				
Mean	0.186	18.05	15.58	4.88
SD	–	29.7	29.76	8.36
P25	–	0.1244	0.11435	0.157
P50	–	0.176	1.012	0.1635
P75	–	21.25	31.0475	4.55
IL12 (pg/ml)				
Mean	2.93	20.1	15.39	16.14
SD	3.966	39.377	18.07	22.08
P25	0.1259	0.24	0.21	0.34
P50	2.93	0.56	12.74	0.35
P75	5.735	39.95	30.58	33.7

Comparing the analyses of cytokine production during LPS stimulation of AMs, revealed the following overall concentrations as depicted in Table 1: IL-1β (0.25–200 pg/10^6^ cells); IL-2 (0.1–7 pg/ml) and IFN-γ (5 pg/ml). An increase in the amount of GM-CSF (100 pg/ml), TNF-α and IL-6 (400–600 pg/10^6^ cells) were found. Moreover, IL-8 was secreted in the nanogram range (3.5 ng/10^6^ cells). Compared to control levels, the pro-inflammatory cytokine secretion was decreased in all three surfactant groups after 24 h exposure. On the other hand, anti-inflammatory cytokines, IL-10 and IL-12, were also seen to be secreted at low levels under LPS-stimulated conditions, but exhibited the opposite nature to the pro-inflammatory cytokines by increasing in secretion when exposed to surfactants.

## Discussion

The use in pulmonary surfactant replacement therapy as a standard care for premature infants with respiratory distress syndrome is well established. However, in addition to its function to modulate surface tension at the air-liquid interface, pulmonary surfactant also have immune modulatory properties. Regrettably, the exact mechanisms by which exogenous surfactants modulate the inflammatory cascade in AMs are still unclear, thus making it important to study the complex interplay among the inflammatory cytokines (inducer or suppressor effects of one cytokine against another) in order to eventually unravel its involvement in inflammatory pulmonary conditions. This prompted us to compare the potential immune modulatory effects of the synthetic pulmonary surfactant Synsurf® on AMs with that of the natural extracted surfactants Curosurf® and Liposurf®.

Due to the small sample size of healthy patients, a standardised cell culture concentration was maintained as to keep statistical integrity regarding pool size. Under our in vitro experimental conditions, mindful consideration was given during the fractionation [3] and handling processes of AMs when we interpreted our results. This was necessary as other researchers showed that macrophage adherence in itself to cell culture dishes, results in the production and release of certain inflammatory mediators [4, 5].

We found a low relationship between LPS stimulated release of IL-1β, IL-2, IL-6 and IL-8 and their spontaneous secretion during prolonged exposure to LPS in combination with surfactant. Synsurf® displayed an approximate two-fold decrease in IL-1β release (97.60 pg/ml) compared to control levels (176.45 pg/ml) whereas both natural surfactants displayed minimal decreases. After 24 h, TNF-α levels decreased within the cell supernatant of the surfactant treated AMs. The Curosurf® (120.73 pg/ml) and Liposurf® (110.01 pg/ml) groups displayed an approximate four-fold decrease in TNF-α release compared to control levels (462 pg/ml); whereas, Synsurf® (56.87 pg/ml) displayed a much larger, eight-fold, inhibitory effect on stimulated TNF-α release. TNF-α and IL-1β release by macrophages are “acute response” cytokines that promote neutrophilic and eosinophilic inflammation. Although they are not directly chemo-attractive agents, they may directly or indirectly stimulate the upregulation of relevant secondary cytokines and cell adhesion molecules. The increased presence of TNF-α and IL-1β may synergistically amplify the expression of IL-6 and IL-8 [6]. This being said, it has been seen that peak TNF-α and IL-1β AM release occurs between approximately 8–12 h after initial exposure [7]. This may explain the rather low LPS stimulated levels of these cytokines at 24 h compared to that of their downstream-regulated cytokines IL-6 and IL-8 (although both IL-6 and IL-8 are decreased within all three the surfactant groups) which may reach their peak concentrations much later. IL-6 was also found to be at much lower levels compared to IL-8. A previous study showed a similar phenomenon and elucidated the relationship between basal secretion and initial spontaneous release for IL-8, IL-6 and TNF- α in culture; once again highlighting the significant cell adherence effect and its subsequent stimulus on cytokine secretion [5].

Both natural pulmonary surfactants decreased secretory levels of IL-2 whereas cell supernatant levels of IL-2 where increased when exposed to the synthetic pulmonary surfactant Synsurf®. It is important to note that IL-2 activates several different pathways that mediate the flow of mitogenic and survival-promoting signals [8]. Moreover, IL-2 induces the JAB/suppressor of cytokine signalling 1 (JAB/SOCS1/SSI-1) that in turn inhibits IL-2 signalling [9]. SOCS1 is involved in the negative regulation of cytokines and initiates an anti-inflammatory response via the regulation of homeostasis. Thus indicating that IL-2 also has anti-inflammatory properties, as does other pro-inflammatory cytokines, such as IFN-γ [10]. Levels of IFN-γ secretion decreased almost 3-fold when AMs were exposed to Curosurf® and Synsurf®; however, only an approximate 2-fold decrease in the presence of Liposurf® was witnessed. IFN-γ is important for the clearance of bacteria by recruiting many other cells (e.g. NK cells), but some reports indicate that IFN-γ is crucial for the cell cycle arrest in macrophage cell cycles that provides a survival signal, and others suggest that it serves as a pro-apoptotic signal. There have been many advances in the understanding of cell cycle control and apoptosis within isolation; however, the inter-relationship between these intimately related processes concerning protective immunity and immunopathology are yet to be fully understood [11].

## Conclusion

There are few studies found in literature on BALs in healthy children as the procedure ethically refers to respiratory problems. This study approached the problem by collecting samples from healthy paediatric patients undergoing fiberoptic bronchoscopy for various reasons without evidence of respiratory tract infections similar to that of previous studies. Our study provides the first data on healthy BALs among South African children without inflammatory pulmonary conditions and the subsequent effect of exogenous surfactant on BAL inflammatory cellular components.

## Data Availability

The data used and analysed during the current study are available from the corresponding author on reasonable request.

## Abbreviations

AM’s: Alveolar macrophages
BAL: Bronchoalveolar lavage
GM-CSF: Granulocyte macrophage colony stimulating factor
IFN-γ: Interferon gamma
IL: Interleukin
LPS: Lipopolysaccharide
TNF-α: Tumour necrosis factor alpha

## References

[1] Connett GJ (2000). Bronchoalveolar lavage. Paediatr Respir Rev.

[2] Van Zyl JM, Smith J, Hawtrey A (2013). The effect of a peptide-containing synthetic lung surfactant on gas exchange and lung mechanics in a rabbit model of surfactant depletion. Drug Des Devel Ther.

[3] Ferro TJ, Kern JA, Elias JA, Kamoun M, Daniele RP, Rossman MD (1987). Alveolar macrophages, blood monocytes, and density-fractionated alveolar macrophages differ in their ability to promote lymphocyte proliferation to mitogen and antigen. Am Rev Respir Dis.

[4] Rolfe MW, Kunkel SL, Rowens B, Standiford TJ, Cragoe EL, Streter RM (1992). Suppression of human alveolar macrophage-derived cytokines by Amiloride. Am J Respir Cell Mol Biol.

[5] Losa García JE, Rodriguez FM, De Cabo MR, Salgado MJ, Losada JP, Villarón LG (1999). Evaluation of inflammatory cytokine secretion by human alveolar macrophages. Mediat Inflamm.

[6] Ishii H, Fujii T, Hogg JC, Hayashi S, Mukae H, Vincent R (2004). Contribution of IL-1β and TNF-α to the initiation of the peripheral lung response to atmospheric particulates (PM10). Am J Phys Lung Cell Mol Phys.

[7] Kerecman J, Mustafa S, Vasquez M, Dixon P, Castro R (2008). Immunosuppressive properties of surfactant in alveolar macrophage NR8383. Inflamm Res.

[8] Benczik M, Gaffen SL (2004). The interleukin (IL)-2 family cytokines: survival and proliferation signaling pathways in T lymphocytes. Immunol Investig.

[9] Sporri B, Kovanen PE, Sasaki A, Yoshimura A, Leonard WJ (2001). JAB/SOCS1/SSI-1 is an interleukin-2–induced inhibitor of IL-2 signalling. Blood..

[10] Bachmann MF, Kopf M (2002). Balancing protective immunity and immunopathology. Curr Opin Immunol.

[11] Schroder K, Hertzog PJ, Ravasti T, Hume DA (2004). Interferon-γ: an overview of signals, mechanisms and functions. J Leukoc Biol.

